# Tina: A diffusion neural network for generating personalized AI models from text prompts

**DOI:** 10.1016/j.patter.2026.101571

**Published:** 2026-05-29

**Authors:** Zexi Li, Lingzhi Gao, Dongqi Cai, Nicholas D. Lane, Chao Wu

**Affiliations:** 1 Zhejiang University, Hangzhou 310027, China; 2 Department of Computer Science and Technology, University of Cambridge, Cambridge CB3 0FD, UK; 3 Knowin AI, Shenzhen 518057, China; 4 School of Intelligence Science and Technology, Nanjing University, Suzhou 215163, China

**Keywords:** generative AI, text-to-model generation, parameter generation, personalization

## Abstract

Generative artificial intelligence (GenAI) has advanced rapidly across modalities, from text-to-text large language models to text-to-image and text-to-video diffusion models. Here, we investigate text-to-model generation: whether GenAI can map semantic task descriptions to functional neural network parameters for personalized classification. We present Tina, a text-conditioned neural network diffusion model that leverages a diffusion transformer conditioned on contrastive language-image pre-training (CLIP)-embedded task descriptions. Tina generates high-quality personalized classifiers across domains, including natural and medical images, from text prompts at inference time. We demonstrate that Tina achieves both in-distribution and out-of-distribution personalization, supports zero-shot/few-shot image prompts, generalizes to unseen classes, and scales to more complex tasks. Tina establishes text-to-model GenAI as a promising paradigm for on-demand personalization and offers a new channel for human-AI interaction through natural-language instructions.

## Highlights

• Tina generates functional neural networks from text prompts on demand
• Text-to-model GenAI enables personalized classification across domains
• Tina generalizes to unseen tasks and classes via CLIP-aligned diffusion
• A new paradigm for human-AI interaction through natural language

## The bigger picture

Artificial intelligence is integral to modern science, yet prevailing models are typically large and general-purpose. Adapting these systems for personalized needs, such as specialized medical diagnostics or lightweight ecological classifiers, requires deep technical expertise and extensive computing resources. This creates a significant barrier that limits the accessibility of customized AI tools for non-expert users. This work explores a shift in human-AI interaction by moving from generating content to directly generating functional AI models. By translating natural-language instructions into working neural network parameters, users can simply describe their desired task. The system then automatically constructs a compact, specialized model ready for quick deployment on everyday devices. The implications of this paradigm extend beyond image classification and pave the way for a more democratized AI ecosystem. It empowers scientists and individuals without coding expertise to build customized AI tools on demand. Ultimately, this approach can accelerate scientific discovery and facilitate the creation of highly tailored AI applications across diverse fields, making the development of specialized AI as accessible as writing a simple sentence.

## Graphical abstract

**Figure:**
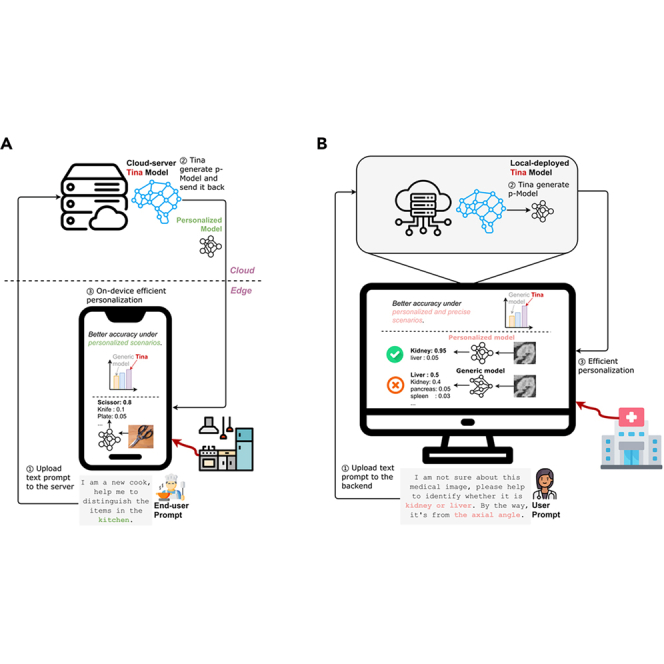

## Teaser

Li et al. introduce Tina, a text-to-model generative AI that produces personalized neural network classifiers from natural-language descriptions. Powered by a conditional diffusion transformer with CLIP embeddings, Tina generates lightweight, task-specific models across diverse domains, including medical imaging, and generalizes to unseen tasks, offering non-expert users a direct channel to create customized AI tools.

## Introduction

Generative artificial intelligence (GenAI)^1^^,^^2^^,^^3^^,^^4^ has been flourishing in different aspects of human life, and people can simply generate content from natural-language text prompts.^5^^,^^6^^,^^7^^,^^8^^,^^9^^,^^10^ Large language models,^5^^,^^11^ such as GPT-4, have especially shown emergent intelligence^12^ in language knowledge through text-to-text transformation.^5^^,^^11^^,^^13^^,^^14^ Besides, recent progress in text-to-image (e.g., stable diffusion)^6^^,^^8^^,^^15^^,^^16^^,^^17^ and text-to-video (e.g., Sora)^7^^,^^18^ diffusion models has enabled them to generate high-fidelity media that mirror the physical world.^6^^,^^7^^,^^8^^,^^15^^,^^16^^,^^17^^,^^18^^,^^19^ These advancements map the meaning of human language to a vast body of world knowledge. In this paper, we take a step further by exploring whether generative models can translate semantic task descriptions into functional neural parameters. We propose and investigate text-to**-**model generation, a new paradigm in which GenAI directly generates functional AI model parameters from a user’s text prompt, a direction also explored in concurrent work on prompt-to-weights generation,^20^ specifically to address the growing and critical need for personalization. In this paper, we study this paradigm in the setting of personalized image classification, where personalization corresponds to generating models for class-subset tasks.

**Figure 1:**
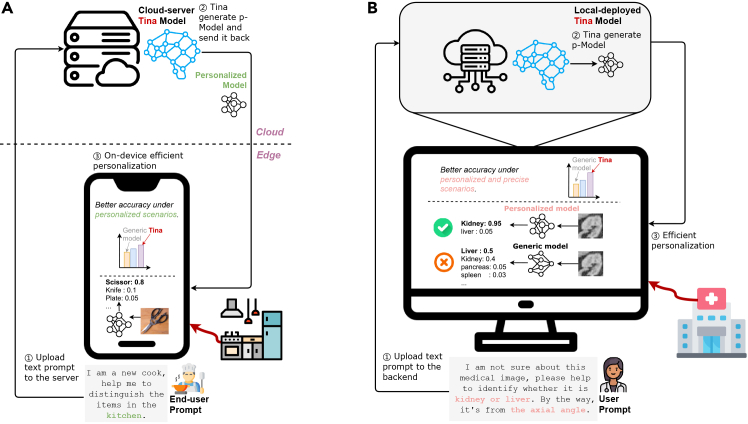
Personalized application scenarios of text-to-model generative AI (A) Text-to-model generative AI can serve for efficient personalized scenarios for end users under edge-cloud collaborations. In the example, an end user can prompt for a kitchen-item classifier, and Tina (our text-to-model generative AI) can generate a lightweight personalized model and deploy this model on the edge for efficient inference. (B) Text-to-model generative AI can serve for scenarios that require more precise prediction, e.g., medical image classification. In the example, users can locally deploy Tina and generate a personalized model when the generic model does not work well under certain scenarios.

The demand for personalization is widespread, spanning from everyday consumer applications to high-stakes professional domains. Current AI systems are often large and all-purpose, which can be inefficient or inadequate for specific user needs. For instance, consider the scenario of an amateur cook (Figure 1A) who requires a simple tool to distinguish a few items in their kitchen. A large, general-purpose vision model would be unnecessary and computationally expensive on a device such as a smartphone. A more effective solution would be an on-demand, lightweight model tailored to that specific environment. In contrast, in scenarios requiring extreme precision, such as medical diagnostics (Figure 1B), a generic model may fail when faced with specialized data, such as distinguishing between organ tissues from a specific imaging angle. A medical professional could instead prompt an AI to generate a highly specialized and precise model to aid their diagnosis, improving accuracy where it matters most. These scenarios highlight a clear need for a way to dynamically create customized and personalized AI models tailored to an individual’s unique context, knowledge, and goals.

To realize text-to-model generation, we propose Tina, a text-to-model GenAI designed for on-demand personalization. Tina treats model generation as a conditional diffusion process, much like how diffusion models denoise random pixels into a coherent image. It is trained on pairs of model parameters and their corresponding task descriptions. By conditioning a diffusion transformer (DiT)^19^ with text and image embeddings from contrastive language-image pre-training (CLIP),^21^ Tina learns to map natural-language prompts to the complex parameter space of neural networks. Our study focuses on a practical personalization scenario initially termed “train-once-for-all,”^22^ in which a single, comprehensive training process enables the later generation of countless specialized models. While the number of possible personalized task combinations is extremely large (e.g., $\left(\begin{array}{l} 100\\ 10 \end{array}\right)\approx 1.73\times 10^{13}$ sub-tasks for a 100-class dataset), we surprisingly find that Tina can generalize effectively from a remarkably small training dataset (∼1,000 data points) to generate models for unseen tasks and even unseen classes.

Our analyses demonstrate that Tina achieves both in-distribution (ID) and out-of-distribution (OOD) personalization. Leveraging the vision-language alignment of CLIP, Tina can accept not only text but also images as prompts, enabling few-shot and zero-shot generalization. More precisely, the semantic prior comes from CLIP’s embedding space, while Tina learns a non-trivial mapping from that semantic space to neural parameter space. We showcase its practical utility in real-world scenarios, including the generation of fine-grained models for medical image classification.^23^^,^^24^^,^^25^^,^^26^^,^^27^^,^^28^ This work investigates the capacity of GenAI to understand and generate AI itself, establishing text-to-model as a promising research field and a powerful tool for creating truly personalized and collaborative AI. In this paper, we focus on the setting of personalized image classification and leave broader forms of text-to-model generation beyond classification to future work.

This ability to generate models from text creates a new, more direct channel for human-AI interaction. More than a technical tool, text-to-model GenAI allows us to rethink how people can participate in the creation of AI. It can help people, including non-experts, develop customized models using simple natural-language instructions. This opens the door for a more human-centered AI environment where GenAI facilitates collaboration and creates social good for users. For the science community, this approach offers a powerful new method for research. As shown in this paper’s medical example, researchers can use text-to-model GenAI to generate specialized tools for science discovery and scientific services. We believe this work helps pave the way for a future in which AI models are not just used by people but co-created with them.

## Results

### Framework overview

We present Tina, a text-conditioned neural network diffusion model for train-once-for-all personalization. Generally, Tina consists of DiT and CLIP encoders for generating personalized models (p-Models) from text prompts. During training, we use the CLIP text encoder to encode texts, and due to the alignment of image and text in CLIP, during inference, Tina can also take images as prompts by utilizing the CLIP image encoder. Additionally, we devise an effective data augmentation approach to enable training Tina under limited samples. We also propose a classification sequence padding strategy to enable Tina to generate models with different class lengths for further personalization.

### Experimental setups

Our evaluation spans three standard natural-image benchmarks (Mini-ImageNet, CIFAR-100, and Caltech-101) and a medical benchmark (MedMNIST), demonstrating cross-domain behavior while keeping the task form fixed to classification.

#### Datasets and p-Models

We used four datasets to conduct experiments, including three commonly used datasets in computer vision (Mini-ImageNet,^29^^,^^30^ CIFAR-100,^31^ and Caltech-101^32^) and one medical dataset of medical image recognition (MedMNIST^27^^,^^28^). Mini-ImageNet is a subset of the ImageNet dataset, primarily used for few-shot learning tasks. CIFAR-100 is a popular benchmark dataset for image classification tasks. Each class contains 600 images, divided evenly into 20 superclasses and 100 classes. Caltech-101 is a dataset for object recognition that features diverse images with varied resolutions and quality. It includes 101 categories, each containing 40–800 images, offering a wider range of objects and scenes than CIFAR-100 and Mini-ImageNet. MedMNIST is a collection of standardized, pre-processed medical image datasets, designed to be lightweight and benchmark ready for diverse biomedical image analysis tasks. We used OrganAMNIST, OrganCMNIST, and OrganSMNIST datasets in MedMNIST for organ classification. For the images with different resolutions, we resized them to 32 × 32 for unified modeling. The personalized tasks were crafted by selecting 10 classes out of the 100/101 total classes. Unless mentioned otherwise, the number of p-Models (i.e., personalized tasks) used to train Tina is 1,000.

We used two architectures for p-Models: a simple convolutional neural network (CNN) (dubbed “CNN”) and ResNet-20 (dubbed “ResNet”). The CNN architecture follows Peebles et al.,^33^ which consists of 2 layers, and the number of parameters is approximately 5,000. We used all the parameters of CNN as the input and output for Tina. But for ResNet, the number of parameters is nearly 272,000, which is too large for Tina’s generation. Thus, we explored partial parameter generation following Wang et al.^34^ We only personalized the classifier layers for parameter generation, nearly 640 parameters.

For more details about data preparation and p-Models, please refer to the supplemental information.

#### Compared baselines

We followed the baselines used in the original paper of train-once-for-all personalization.^22^ As described in the dataset preparation subsection, we used the generic model trained in stage 1 as a baseline, showing the performance without any personalization. Further, we compared the classifier selection method described in the strong baselines subsection, which serves as a strong baseline for personalization.^22^ Also, TAPER, the proposed method in train-once-for-all personalization,^22^ learns to aggregate several customized models instead of directly generating the models.

#### Evaluation metrics

For Table 1, we compared ID ability and OOD ability, as elaborated in the dataset preparation subsection. For other tables and figures, we reported the OOD personalization as p-Acc (personalized accuracy). It is notable that for every setting, we tested personalization performance across more than 100 tasks (i.e., 100 independent trials) and report the average scores in the tables and figures. Each task includes more than 1,000 testing image samples. Therefore, the evaluation measurement is statistical, representative, and fair for the compared methods. The presented results are in percentages (%).

**Table 1:** Main results on three computer vision datasets

Dataset	Mini-ImageNet		CIFAR-100		Caltech-101		Average	
p-Models	CNN	ResNet	CNN	ResNet	CNN	ResNet	CNN	ResNet
**In-distribution personalization**								

Generic model	19.76	39.32	28.72	51.24	29.14	47.95	25.87	46.17
Classifier selection	51.74	71.49	64.83	84.01	56.07	74.75	57.55	76.75
TAPER	52.16	65.50	67.71	75.12	58.48	77.92	59.45	72.85
**Tina**	**54.08**	**74.99**	**68.35**	**86.46**	**58.69**	**78.36**	**60.37**	**79.94**

**Out-of-distribution personalization**								

Generic model	18.55	39.80	29.88	52.24	29.14	50.56	25.86	47.53
Classifier selection	51.02	72.47	64.15	83.94	56.44	76.03	57.20	77.48
TAPER	51.64	67.03	66.85	72.30	58.93	79.65	59.14	72.99
**Tina**	**53.31**	**75.34**	**67.14**	**86.63**	**59.27**	**79.69**	**59.91**	**80.55**

The best results are in bold.

#### Hyperparameters

The detailed hyperparameters can be found in the supplemental information.

### Performance evaluation

In Table 1, we evaluated the performance of our proposed method, Tina, against several baseline methods, including generic model, classifier selection, and TAPER, across various datasets and model architectures for the task of train-once-for-all personalization. It is found that the generic model has inadequate performance, validating the need for personalization techniques. For the personalization methods, the results demonstrate that Tina consistently outperforms all baseline methods across both ID and OOD personalization scenarios. Though Tina is a text-to-model foundation model, it is worth noting that it shows intelligence in personalization under limited data (nearly 1,000 samples). Specifically, for ID personalization, Tina achieves significant improvements with an average score of 79.94, surpassing the next-best method, classifier selection, by a margin of 3.19. Similarly, for OOD personalization, Tina leads with an average score of 80.55, an obvious increase of 2.78 over the second-best-performing method. In addition, TAPER has inferior performance compared with Tina, showing the advantages of Tina as a generative model in parameter generation. TAPER only learns to merge the expert models, whereas Tina learns to directly generate the parameters (see Table S2 for further analysis demonstrating that Tina generates diverse parameters rather than merely memorizing training data).

We verified whether Tina is effective for larger and more complex p-Models in Table 2.

**Table 2:** Results on larger p-Models (ViT-B/32)

Dataset	CIFAR-100	Caltech-101
**In-distribution personalization**		

Classifier selection	95.07	96.33
TAPER	95.25	96.32
**Tina**	**95.45**	**97.15**

**Out-of-distribution personalization**		

Classifier selection	94.78	96.08
TAPER	94.96	96.15
**Tina**	**95.15**	**96.72**

We used ViT-B/32 pretrained by CLIP, and Tina generates the personalized layers in ViT as described for ResNet. The results are promising: our Tina can achieve up to 97.15% accuracy in personalization when using the ViT-B/32 backbone pretrained by CLIP and also consistently outperforms the baselines. It showcases the scalability and potential of Tina to be adopted in trending and state-of-the-art architectures and to reach state-of-the-art performance in personalization.

### Applications of Tina

#### Results under medical datasets

We conducted experiments using the OrganAMNIST, OrganCMNIST, and OrganSMNIST datasets in the MedMNIST benchmark.^27^^,^^28^ These datasets are derived from computed tomography (CT) images and represent axial, coronal, and sagittal views, respectively. They are used for multi-class classification involving 11 different human organs.

We treated these three datasets as a single large dataset with multiple domains, where the different organ views correspond to different domains. A CNN model was used for full parameter generation. In the first stage, we trained a generic model on the combined overall dataset from all three domains. For the personalization process, tasks are configured as 5-way classification problems, each originating from a classification task within one specific domain. In the second stage, we fine-tuned the generic model on the corresponding dataset to obtain a p-Model for training with Tina. We measured the methods under two metrics: top-1 accuracy and area under the curve (AUC).

For each domain, we trained 200 p-Models, resulting in a total of 600 p-Models for training Tina. For the text prompts, we further specified the format as “domain + class name” (e.g., “axial bladder”) to guide Tina. The goal is for Tina to generate models capable of solving classification tasks corresponding to specific domains, conditioned on the provided domain and class names. Tina is expected to learn and distinguish the knowledge of different image views (domains) and generate a p-Model for each specific task. For TAPER, we used the domain as the partitioning criterion. Each domain is fine-tuned from the generic model, resulting in three corresponding expert models.

**Figure 2:**
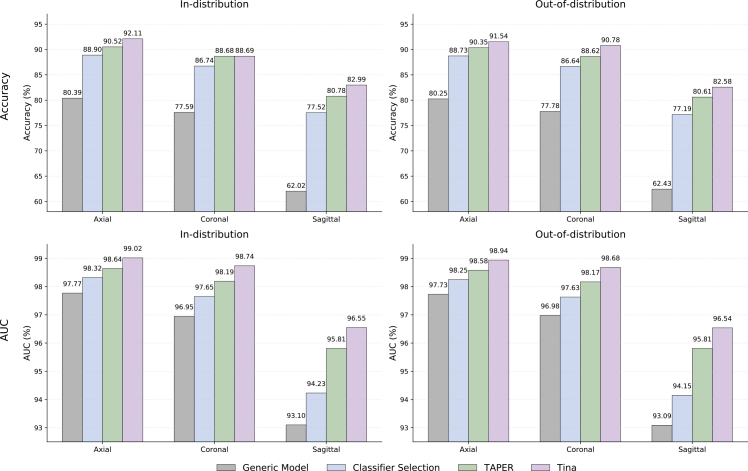
Results under medical datasets MedMNIST “Axial” refers to OrganAMNIST in MedMNIST, which contains CT images from the axial view. Similarly, “coronal” stands for OrganCMNIST from the coronal view, and “sagittal” refers to OrganSMNIST from the sagittal view.

Figure 2 presents the results on medical datasets. It shows that Tina can achieve high performance across domains for both ID and OOD abilities. In particular, Tina excels the best in the Sagittal domain compared with the baselines, and the performance gains may result from its generalization of absorbing the knowledge across different image views. This experiment showcases Tina for broader applications, and it has the potential to enable personalized or precision medicine by taking patient status/description as prompts.

#### Application in personalized federated learning

We further investigate the performance of Tina in edge-cloud distributed personalized training by integrating the algorithm into a personalized federated learning (FL) environment. Specifically, we utilize the CIFAR-100 dataset to construct a heterogeneous data environment with natural feature skew. While the entire distributed system performs classification tasks across 20 superclasses, each user’s local data are derived from different subclasses. This setup simulates a realistic non-independent and identically distributed (non-IID) scenario where each user has distinct data classification requirements.

To simulate realistic distributed environments, we vary the number of clients (*N* ∈ {15,20,25,30}) and the sampling rates (i.e., the ratio of clients participating in aggregation per round). We compare our proposed method, FedAvg + Tina, against several baselines. (1) Standard Baselines: FedAvg^35^ and state-of-the-art personalized algorithms FedRoD^36^ and FedCP.^37^ (2) Per-FedAvg^38^: a meta-learning-based algorithm that customizes p-Models. (3) FedAvg + pre: a strong baseline where clients are initialized with a model pretrained on the entire CIFAR-100 dataset (generic initialization).

In our FedAvg + Tina approach, we employ a “prompt-based FL” paradigm. Tina is first trained on the CIFAR-100 dataset. Then, instead of uploading raw data, each client provides a textual description of their local data distribution. Based on these prompts, Tina generates a specific, personalized neural network initialization (“warm start”) for each client.

The quantitative results are presented in Table 3. The generation of personalized neural networks significantly improves performance. While Per-FedAvg customizes models, it often requires sufficient local data to be effective. Notably, FedAvg + Tina consistently outperforms FedAvg + pre. This performance gap indicates that personalizing the initialization via textual descriptions captures task-specific knowledge more effectively than a high-quality generic initialization. This demonstrates Tina’s ability to address diverse user needs even with limited local data.

**Table 3:** Test accuracy comparison of FedAvg + Tina and baselines on CIFAR-100 under varying client numbers (*N*) and sampling rates

No. of clients (*N*)	15		20		25		30	
**Sampling rate**	**0.6**	**1.0**	**0.6**	**1.0**	**0.6**	**1.0**	**0.6**	**1.0**

FedAvg^35^	41.95	44.11	39.81	43.79	39.36	42.22	39.77	41.44
Per-FedAvg^38^	37.15	35.27	37.63	37.4	35.43	38.62	36.92	39.07
FedRoD^36^	45.38	44.42	42.18	42.41	41.52	41.33	41.13	41.63
FedCP^37^	46.10	46.94	41.75	41.34	40.46	40.66	39.90	40.54
FedAvg + pre	47.50	50.51	46.38	49.03	46.62	48.76	46.61	48.03
**FedAvg + Tina**	**48.17**	**51.69**	**47.53**	**50.87**	**48.02**	**51.08**	**48.14**	**50.94**

**Figure 3:**
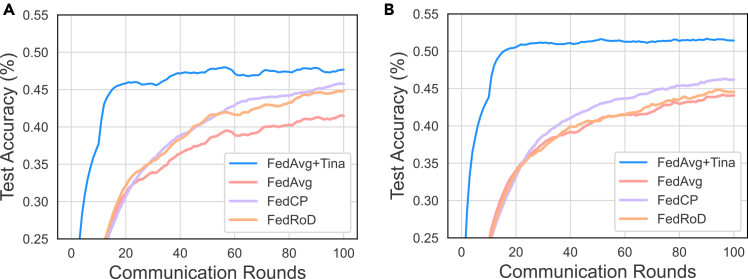
Performance evaluation of FedAvg + Tina versus baselines on CIFAR-100 (*N* = 15) across different sampling rates Left: 0.6. Right: 1.0.

Furthermore, we analyze the convergence behavior in Figure 3. With the personalized parameters generated by Tina, FedAvg converges significantly faster than other FL algorithms. This rapid convergence reduces the number of required communication rounds, thereby decreasing the time for users to obtain a personalized network while guaranteeing performance. The results suggest that Tina-generated parameters encapsulate task-relevant knowledge, allowing clients to quickly adapt to local data. This experiment highlights Tina’s potential to facilitate efficient, privacy-preserving edge-cloud collaborative training without requiring raw data transfer.

### In-depth analysis of Tina

Tina shows great potential for text-to-model generation for personalization. We have made several in-depth analyses to better understand the capabilities and boundaries of Tina, and we will show insights into how Tina learns hyper-level world knowledge, as well as its limitations for future research. Unless mentioned otherwise, we use CIFAR-100 as the dataset for analyses.

#### Scaling studies for Tina

**Figure 4:**
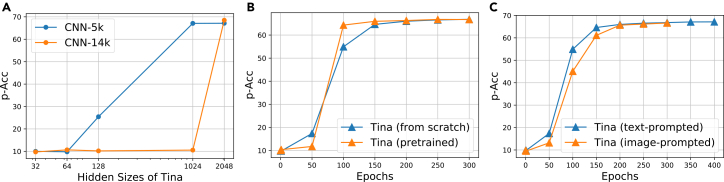
Tina capability analysis with regard to different parameterization and training schemes (A) Scaling the parameters of DiT in Tina. CNN-5K (14K) means the p-Model is a CNN with 5,000 (14,000) parameters. From 152 (hidden size 32) to 789 (hidden size 2,048) M, scaling helps in the emergence of intelligence. (B) Parameter inheritance from pretrained G.pt helps speed up training in the early stages. (C) Training Tina with image-prompted data versus text-prompted data. The text-prompted has faster convergence.

**Figure 5:**
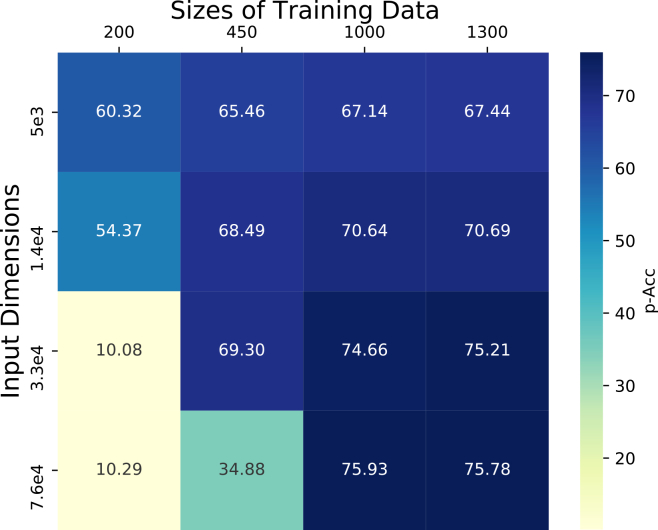
Scaling the input dimensions and training data for Tina

Scaling laws for transformer-based foundation models have shown that increasing model parameters, training data, and computational budget can lead to emergent capabilities. In Figure 4A, we scaled the parameters of Tina by varying the hidden size, ranging from 32 (152 M parameters) to 2,048 (789 M), and we tested two p-Model sizes. It is found that when Tina is small, it fails to generalize, especially when the p-Model has a higher parameter dimension. The intelligence emerges when scaling Tina to larger sizes (e.g., 1,024 or 2,048 hidden sizes), but the scaling effect is saturated when reaching the upper-bound performance of personalization. We also scaled the input and generated dimensions (i.e., p-Model sizes) and the training data in Figure 5. It is found that a larger input dimension is harder to learn and requires larger sizes of training data to converge and generalize. The generalization of Tina can benefit from larger training data, but it has diminishing marginal returns. Generally, larger p-Models, larger training samples, and larger model sizes lead Tina to achieve higher p-Acc, demonstrating the increasing expressive power of Tina with scaling, which is consistent with previous DiT works.^7^^,^^19^^,^^33^ The scaling property indicates the great potential of Tina for more complex and challenging text-to-model scenarios.

#### Parameter inheritance

We verified whether Tina can benefit from pretrained parameters. We inherited the parameters from G.pt’s^33^ checkpoints using the bert2BERT-like method.^39^ From Figure 4B, it is found that parameter inheritance from pretrained models can help Tina to converge faster, but the final p-Accs are similar.

#### Training images as prompts

In the original design of Tina, text is used for the prompts encoded by the CLIP text encoder. We trained Tina with image prompts using a CLIP image encoder, and the results are shown in Figure 4C. For each class, we randomly selected a single image as the prompt. It is found that text-prompted Tina converges faster than the image-prompted one, though the final p-Accs are similar. This is intuitive to understand since texts are known to have higher knowledge density than images^21^^,^^40^ and the class text has richer knowledge representations than a single image.

#### Testing images as prompts

**Figure 6:**
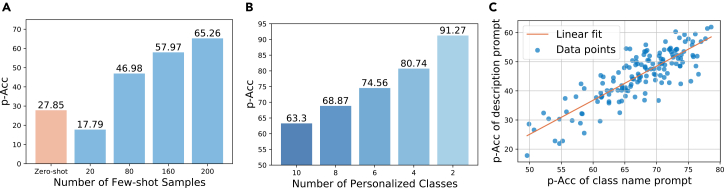
Tina’s capability analysis with regard to different prompt schemes (A) Train text-prompted Tina and verify the zero-shot and few-shot abilities of using images as prompts. (B) The accuracy of p-Models generated by Tina varies with different numbers of classes. Classification sequence padding is used, and the maximal sequence length is 10. (C) Train class-name-conditioned Tina and verify its zero-shot ability on the natural-language descriptions generated by GPT-4.

We trained text-prompted Tina and verified its zero-shot and few-shot abilities on image prompts, and the results are shown in Figure 6A. Due to the alignment between text and images in CLIP, Tina shows zero-shot ability on image prompts. With few-shot fine-tuning on image prompts, Tina can achieve comparable performances to the text-prompted model. We note that the image-prompted ability is important in practical personalization scenarios, as some users may have few images and want a p-Model for those. The images are too few to train a model from scratch, but thanks to the generative power of Tina, we can generate a p-Model from image prompts by utilizing Tina’s vision-language-parameter-aligned knowledge.

#### Varying the number of personalized classes

Without changing architecture, Tina can adapt to any personalized classes within the maximal supported length due to the padding design. In Figure 6B, we tested the p-Models with different numbers of classes, generated by a single Tina. The maximal classification length is 10. It is shown that the generated p-Models achieve higher p-Accs when there are fewer classes, which is consistent with common sense that fewer classes are easier to personalize.

#### How Tina understands world knowledge I: Natural-language descriptions as prompts

In our implementation of Tina, we adopted a simple prompting that uses class names as the text prompts. We verified whether Tina actually learns the knowledge when the prompts are replaced with natural-language descriptions at test time. We generated the language descriptions of classes with the assistance of GPT-4,^41^ and we made sure that the descriptions did not include the original class entities. The exemplars are shown in the supplemental information (Table S1). As shown in Figure 6C, the results reveal that Tina has zero-shot generalization ability when the prompts are unseen language descriptions, though the p-Accs are lower than those of the class-named prompts. It shows that Tina is not just memorizing the class names but also generalizing and understanding the knowledge behind the names and the nuances inherent in the text’s semantics.

#### How Tina understands world knowledge II: Generalization to unseen classes/entities

We divided the CIFAR-100 dataset into two disjoint shards of classes and trained Tina on one shard, then verified its generalization to the unseen classes of another shard. Results in Table 4 showcase that Tina has the intelligence to generalize to unseen classes, while TAPER fails when meeting 100% unseen classes. As a generative model, Tina can understand the hyper-level world knowledge embedded in model parameters as well as text semantics and generate models to predict unseen entities (see Figure S1 for a CLIP text embedding t-distributed stochastic neighbor embedding [t-SNE] visualization showing the semantic overlap between seen and unseen tasks).

**Table 4:** Zero-shot transfer of Tina to unseen classes

Settings	0% unseen classes	20% unseen classes	40% unseen classes	60% unseen classes	100% unseen classes
TAPER	60.27	51.94	42.48	31.45	–
Tina	**62.51**	**55.36**	**49.17**	**42.78**	**30.93**

We test the generalization capability of Tina to unseen classes that have textual similarity to the seen ones.

### Ablation of the design choices of Tina

We performed an ablation study for different design choices of Tina. The ablated designs are the ones different from previous literature, such as our design of classifier augmentation, G.pt’s design of permutation augmentation,^33^ and TAPER’s design of merging text embedding as one.^22^ The results are shown in Table 5. Our classifier augmentation can boost the performance even under small training datasets.

**Table 5:** Ablation study for different design choices of Tina

Designs/datasets	Mini-ImageNet	CIFAR-100	Caltech-101	Average
Without classifier augmentation	32.45	49.61	41.61	41.22
With permutation augmentation	9.88	10.14	10.59	10.20
Merge text embedded as one	10.04	10.35	10.78	10.39
Tina (completed)	**53.31**	**67.14**	**59.27**	**59.91**

Permutation augmentation has negative effects on generating personalized models, and we hypothesize that for Tina’s training data, the p-Models fine-tuned from the same generic model are located in a common loss basin, where permutations will disturb the shared representations. In addition, merging the text embeddings into one will hinder the DiT from learning the sequential classifications, making Tina bad at generalization.

#### Ablation of text prompts

We have performed an in-depth ablation study on the impact of text prompts, as shown in Table 6. It is found that when training and testing use the same kind of text prompts, performance is similar regardless of whether class-name or description prompting is used. However, if the prompt strategies differ between training and testing, the results will degrade, and training in class-name prompts has better transferability and generalization.

**Table 6:** Ablation study of Tina on the impact of text prompts

Training prompt	Testing prompt			
	Class name		Description	
	ID	OOD	ID	OOD
Class name	67.27	67.21	46.93	46.77
Description	42.79	42.58	67.29	67.08

The model is CNN, and the dataset is CIFAR-100.

## Discussion

In this paper, we present Tina, a text-to-model GenAI model for personalized classification. Tina has shown great capability in generating p-Models from text prompts and can generalize to ID and OOD tasks, zero-shot/few-shot image prompts, natural-language prompts, and unseen classes. Tina also supports personalization under different numbers of classes. It is also verified that Tina can improve the performance of personalized classification for realistic medical datasets. Moreover, Tina demonstrates superior computational efficiency with orders-of-magnitude speedup over conventional fine-tuning (Table S3) and maintains competitive robustness under common input corruptions (Table S4).

Despite the merits of Tina, it has some current limitations. One limitation is that a single Tina cannot generate p-Models across different sizes and modalities; in the future, large-scale pretraining for Tina may be promising to reach this goal. In this initial study, we focus on personalized image classification. Extending text-to-model generation to other modalities (e.g., audio or text classifiers) and to broader task families remains future work.

This paper explores the potential of text-to-model GenAI and shows that it can serve personalized scenarios. It is the preliminary research to study whether AI can understand and generate AI, and it helps us better understand and use the techniques of GenAI, such as diffusion models. We hope it will have broader impacts on both the machine learning and fundamental science communities. For the machine learning community, this paper may broaden the definition of text-prompted GenAI and also provide new insights for model personalization. Also, the techniques of text-to-model GenAI can serve in scenarios such as edge AI, where end users only have the resources to prompt a model rather than train it and there is demand for personalization in certain data scenarios. In the future, more advanced techniques, such as functional neural memory frameworks,^42^ can be proposed to extend the boundaries of the current Tina. For the scientific community, researchers may explore more practical applications based on text-to-model GenAI in the future, utilizing the foundation model’s power for scientific discovery and services. As shown in this paper, text-to-model GenAI can serve in scenarios that require more precise predictions, e.g., medical diagnosis. Additionally, we believe text-to-model GenAI can provide a new channel for human-AI interaction: AI can help humans generate more customized and personalized AI models based on their instructions. It can help people rethink how non-expert users can be involved in the development of AI by using natural-language instructions and how GenAI can facilitate a more human-centered AI environment and make social goods for users.

## Methods

### Problem setup

#### Definition of setup

Following previous work,^22^ we consider image classification for train-once-for-all personalization due to the natural personalization requirements of image classification. While the proposed framework is conceptually applicable beyond classification, in this work, we focus on personalized image classification; extending to other modalities and task types is left for future work.

Define a task *k* as classification over a subset of classes $Y_{k}\subset Y$. The goal of personalization is to learn a neural network predictor $f_{\theta_{k}}:X\mapsto Y_{k}$, parameterized by *θ*_*k*_. To handle many tasks at the same time, we further assume we have the task description natural text *t*_*k*_ for $Y_{k}$, which generally describes the classes and styles of $Y_{k}$. We want to build a neural network generator *G*(*t*_*k*_) that, given *t*_*k*_, it will output the model parameters *θ*_*k*_. Specifically, consider using a large-scale dataset with many classes covering Y to learn the personalized-friendly function $f_{\theta_{k}}=G_{\phi }\left(t_{k}\right)$ parameterized by *ϕ*. *G*_*ϕ*_ is learned on a large dataset to generate any p-Model directly from task descriptions, and the setup is called train-once-for-all personalization.^22^ Train-once-for-all personalization has wide applications in a server-user system, where the model generator *G*_*ϕ*_ is learned on the server for personalized cloud services to many future users. We refer to TAPER^22^ for more detailed advantages and usages of train-once-for-all personalization.

#### Strong baselines: Classifier selection and TAPER

##### Classifier selection

For a generic network *f*_*θ*_, we consider that it consists of a feature extractor parameterized by *ψ* with a linear classifier $w=\left[w^{\left(1\right)},\ldots ,w^{\left(\left|Y\right|\right)}\right]$ of |Y| vectors for output predictions over all classes in Y. The generic model is trained on a large dataset, and we want to personalize it for a few-way classification task *k*. One effective method is to build a personalized classifier **w**_*k*_ by selecting only the row vectors in **w** for the relevant classes. Therefore, the p-Model for task *k* is *θ*_*k*_ = {*ψ*,**w**_*k*_}, and this approach is called classifier selection, which serves as a strong baseline.^22^

##### TAPER

We briefly introduce TAPER,^22^ proposed by the original paper on train-once-for-all personalization, and discuss its limitations. The main idea of TAPER is to train several experts (bases) and learn a mixture network to fuse these experts into a p-Model. It has three stages as follows.

• Stage 1: train a generic model on the large dataset.
• Stage 2: divide the dataset into several shards and fine-tune the generic model on each shard, respectively, for specification. Each fine-tuned model can be seen as a domain expert.
• Stage 3: for a given personalized task, learn an multi-layer perceptron (MLP) mixer (i.e., the generator *G*) whose input is the text embedding of the task description and the output is the aggregation weights of the expert models. Then, weighted aggregation is conducted to merge several expert models into a personalized one. Also, the expert models can be fine-tuned during personalization.

TAPER is a learning-to-aggregate method, not a parameter generation GenAI, so it cannot generalize to unseen entities. Also, the MLP mixer only generates aggregation weights rather than parameters, so it has limited generalization and expressiveness. While in our design of Tina, we try to construct an end-to-end text-to-model system that can understand the hyper-knowledge residing in parameters and generalize to unseen tasks, even unseen classes.

### Dataset preparation

We introduce how to conduct datasets for training and testing Tina under personalized scenarios. Classification tasks are used because they provide a clear definition of personalization by sampling the sub-distributions of labels/classes.

#### Training data preparation for Tina

Tina takes the p-Model parameters as training data for diffusion training, and the dataset is conducted in two stages. (1) Stage 1: similar to the initial work of train-once-for-all personalization,^22^ we train a generic model on a large dataset to let the model have a generic capability on all classes. (2) Stage 2: we craft the personalized tasks and fine-tune the generic model on the personalized tasks to obtain the p-Models for Tina’s training. For each personalized task *k*, we select the corresponding task combination of $\left|Y_{k}\right|$ classes out of |Y| classes to craft samples used to train the p-Model and then fine-tune the generic model on the samples to get a p-Model as a data point for training Tina. Each data point for Tina contains the “(task description, p-Model)” pair.

#### Testing data preparation

**Figure 7:**
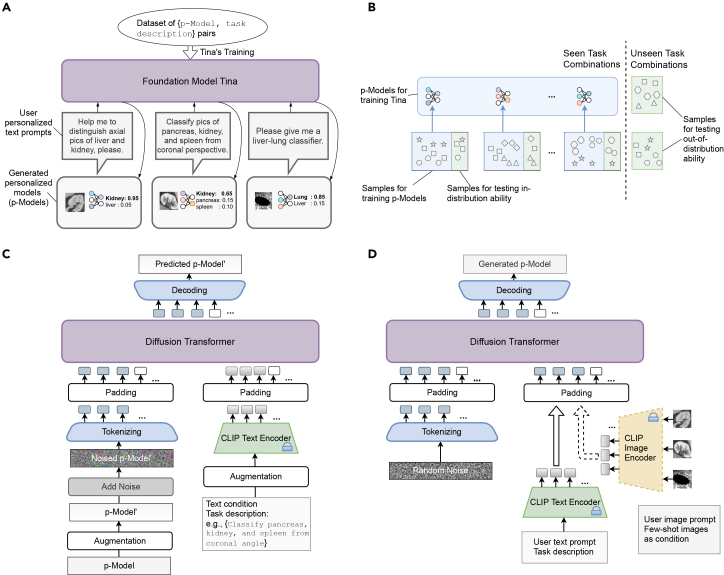
Tina’s training and inference pipelines (A) The overall pipeline and personalization scenario demonstration. Tina is trained on p-Model and task description pairs. After training, users have text descriptions of the desired personalized models. By simply prompting the foundation model Tina, it can generate high-quality personalized models. (B) Description of the training and testing data for Tina. p-Model is short for personalized models. The blue blocks are for training, and the green blocks are for testing/inference. (C) Training framework overview of Tina. The p-Models are firstly augmented by our classifier augmentation strategy and then noised according to the diffusion step. The p-Models are tokenized into chunks of vectors, and optional classification sequence padding is used if the classification length is shorter than the default. The CLIP text encoder is used to encode the users’ text prompts during training. (D) Inference framework overview of Tina. Random noise is tokenized and denoised into parameters of p-Models. Thanks to the vision-language alignment of CLIP, Tina takes both text and visual prompts as diffusion conditions.

The overall demonstration of data partitions can be found in Figure 7B. The blue blocks represent the training data, and the green blocks represent the testing data. For testing, there are two kinds of evaluation metrics. (1) ID ability: the personalized task combinations are seen during the training of the GenAI Tina, and Tina generates p-Models that will be tested on the testing samples of each seen task. (2) OOD ability: the task combinations are unseen during Tina’s training, and Tina directly generates p-Models from the task prompts (the text descriptions).

### Proposed Tina: Text-conditioned neural network diffusion model

#### Architecture and training objective

The framework overview is provided in Figure 7. We use diffusion models as the generative model and follow the main architecture of G.pt^33^ that uses a DiT as the backbone. Analogous to the optimization process that takes random initialization as input and outputs trained models, the diffusion process takes noise as input and gradually denoises to recover the original distributions. Previous works have shown the rationale of neural network diffusion.^33^^,^^34^^,^^43^^,^^44^^,^^45^^,^^46^ We choose DiT as the backbone because it can be easily scaled up and is shown to have great generalization and expressiveness. We use signal prediction for the diffusion process and inherit the architecture of GPT-2^14^ as the transformer. The used text encoder is the pretrained ViT-B/32 in CLIP.^21^

##### Training objective

Denote the training set of Tina as K, where each piece of data is a (task description, p-Model) tuple, notated as (*t*_*k*_,*θ*_*k*_) for task k∈K. We denote the CLIP text encoder by *T*, and given the task description *t*_*k*_, the text embedding is *T*(*t*_*k*_). The text encoder is frozen during training.

Our DiT model *G*_*ϕ*_ takes two vectors as input: the text embedding *T*(*t*_*k*_) as a condition and the noised p-Model parameter vector $\theta_{k}^{j}$, where *j* ∈ [*J*] denotes the timestep in the diffusion forward noising process. The learning objective of diffusion is to minimize the simplified variational lower bound, which reduces to predicting the denoised p-Model parameters:

(Equation 1) $$\min_{\phi }L\left(\phi \right)=\sum_{k\in K}\sum_{j\in J}|\left|\theta_{k}-G_{\phi }\left(T\left(t_{k}\right),\theta_{k}^{j},j\right)\right|{|}_{2}^{2}\text{,}$$

where the timestep *j* is embedded in DiT by frequency-based encoding.^47^ The detailed training procedure is shown in Algorithm 1. We use denoising diffusion probabilistic model (DDPM) sampling^15^; add Gaussian noise depicted by the $\bar{\alpha }$ to *θ*_*k*_ and gradually denoise it.

Algorithm 1 **Tina training**

1: **Input:** number of training iterations *N*_iter_, p-Model dataset $K={\left\{\left(t_{k},\theta_{k}\right)\right\}}_{k=1}^{K}$, Tina, diffusion process length *J*, diffusion cumulative variance schedule $\bar{\alpha }$.

2: **Initialize:** learnable parameters *ϕ* for *G*

3: **for** *i* = 1,2, …,*N*_iter_ **do**

4:  ▷ # Sample a mini-batch of data

5:  $\left(t_{k},\theta_{k}\right)∼K$

6:  ▷ # Noise p-Model parameters

7:  *j*∼*U*({1, …,*J*})

8:  $\theta_{k}^{j}∼N\left(\sqrt{\bar{\alpha_{j}}}\theta_{k},\left(1-{\bar{\alpha }}_{j}\right)I\right)$

9:  ▷ # Compute the predictions

10:  ${\hat{\theta }}_{k}\leftarrow G_{\phi }\left(T\left(t_{k}\right),\theta_{k}^{j},j\right)$

11:  ▷ # Compute the loss

12:  $\text{loss}\leftarrow |\left|{\hat{\theta }}_{k}-\theta_{k}\right|{|}_{2}^{2}$

13:  ▷ # Update DiT’s parameters

14:  *ϕ*_*i*+1_←update(loss;*ϕ*_*i*_).

15: **end for**

#### Design details

We elaborate on the design details of Tina.

##### Parameter tokenization

For p-Model’s parameters *θ*_*k*_, we first flatten all the parameters into a 1D vector and chunk/tokenize the parameters within each layer. If the chunk size is *M* and the number of parameters in a certain layer is *N*, then there will be *ceil*(*N*/*M*) tokens for that layer. For some layers smaller than *M*, the whole layer is a token.

##### Text embedding

Assume the personalized task is a classification task that has $c=\left|Y_{k}\right|$ classes. The task description *t*_*k*_ is an ordered list of the classes’ text descriptions, of which the simplest form is the class entity, e.g., “telephone” and “rabbit.” The generated p-Model is expected to have the correct predictions in the same order as *t*_*k*_. In other words, we need Tina to learn the correct classifier orders as the text prompts, which is sequence-to-sequence modeling. Therefore, unlike TAPER, which averages the class embeddings into one, we make every class description a token by using the CLIP text encoder and concatenate them in order with positional encoding.

##### Encoding and decoding of tokens

We use linear layers as encoders for mapping the parameter tokens and text embedding tokens to the hidden size of DiT. Each token has a different linear layer without weight sharing. The decoders are similar to encoders, which use linear layers, and the encoders transform the transformer’s hidden size back to the p-Model’s parameter dimension. Between the encoders and decoders, there are transformer attention layers similar to those in GPT-2.

##### Data augmentation

In previous work,^33^ the permutation invariance property^48^^,^^49^^,^^50^ is utilized for data augmentation by randomly permuting the neurons without changing the function. However, in our scenario, we find that this augmentation will even impede training. We hypothesize that the p-Models are fine-tuned from the same generic model, so they may lie in the same or close loss landscape basins; as a result, permutation augmentation will disturb network representations and impair Tina training. Further, we develop an effective classifier augmentation strategy to speed up Tina training with limited data by randomly permuting the order of classes in the task description and the order of the corresponding classifier vectors during training. This data augmentation improves sample diversity and helps the DiT better learn the description-to-classifier sequence modeling in a position-aware manner.

##### Parameter inheritance

In previous work,^33^ the authors released a pretrained checkpoint of G.pt, which is also DiT for parameter generation. G.pt is pretrained on large datasets of optimization checkpoints. Although it has different conditions, designs, and scenarios from ours, we explore whether we can inherit some parameters from the pretrained checkpoints to speed up and boost training. Given that the model sizes and architectures are different, we use a strategy similar to bert2BERT^39^^,^^51^^,^^52^ to inherit parameters.

##### Classification sequence padding

We study how to incorporate more personalized settings where diverse users request tasks with different numbers of classes. In language models,^11^^,^^53^ padding is used to enable sequence-to-sequence learning with different input and output lengths. Inspired by this, we use the padding technique to enable the description-to-classifier sequence of different classification lengths. Specifically, if the user’s number of classes is smaller than the maximal length, we pad missing classes with the token “<->” in the task description list and mask the corresponding classifier vectors with zero-like tensors. We denote this strategy as classification sequence padding, and Tina can learn to adapt to any number of classes within the maximal length.

## Resource availability

### Lead contact

Further information and requests for resources should be directed to and will be fulfilled by the lead contact, Zexi Li (zexi.li@zju.edu.cn).

### Materials availability

This study did not generate new unique materials.

### Data and code availability

• All code required for reproducing the results of this work is available on a public repository https://github.com/aoliliao/Tina and has been archived at Zenodo.^54^ Trained model checkpoints are available on Hugging Face.^55^ We also provide sufficient details in the methods and supplemental information for implementing experiments in this work.
• The datasets used in this paper are all publicly released. The Mini-ImageNet dataset^29^^,^^30^ is available at https://huggingface.co/datasets/timm/mini-imagenet. The CIFAR-100 dataset^31^ is available at https://www.cs.toronto.edu/∼kriz/cifar.html. The Caltech-101 dataset^32^ is available at https://data.caltech.edu/records/mzrjq-6wc02. The MedMNIST datasets^27^^,^^28^ (OrganAMNIST, OrganCMNIST, and OrganSMNIST) are available at https://medmnist.com/. The usage of these datasets in this paper is permitted under their licenses.

## Acknowledgments

This work was supported by the National Key Research and Development Project of China (2021ZD0110505), the Zhejiang Provincial Key Research and Development Project (2023C01043), Laboratory for Statistical Monitoring and Intelligent Governance of Common Prosperity, Zhejiang GongShang University, the 2024 Innovation Fund Project of the Engineering Research Center of Digital Learning Technology Integration and Application, Ministry of Education of China, and the Academy of Social Governance, Zhejiang University. This research was also supported by the following entities: The Royal Academy of Engineering via DANTE (a RAEng Chair); the European Research Council especially the REDIAL project; and SPRIND under the composite learning challenge.

## Author contributions

Z.L. and L.G. contributed to the method; Z.L. and L.G. contributed to the main experiments; Z.L., L.G., and D.C. contributed to the writing, presentation, and some supplementary experiments; and C.W. and N.D.L. provided mentorship in method, experiments, and writing.

## Declaration of interests

The authors declare no competing interests.

## Declaration of generative AI and AI-assisted technologies in the writing process

During the preparation of this work, the authors used GPT-4 to generate natural-language descriptions of class names for experimental evaluation. After using this tool, the authors reviewed and edited the content as needed and take full responsibility for the content of the publication.

## Supplemental information

Document S1. Figures S1, Tables S1–S4, and supplemental methods

Document S2. Article plus supplemental information
