# Supplementary material for: Tina: A diffusion neural network for generating personalized AI models from text prompts
Source: Patterns (N Y). 2026 May 29;7(7):101571. doi: 10.1016/j.patter.2026.101571 (PMC13366521; doi:10.1016/j.patter.2026.101571)
Supplement: Document S1. Figures S1, Tables S1–S4, and supplemental methods [file mmc1.pdf]

Patterns, Volume *7*

Supplemental information

Tina: A diffusion neural network for generating personalized AI models from text prompts

Zexi Li, Lingzhi Gao, Dongqi Cai, Nicholas D. Lane, and Chao Wu

S1 Supplemental Methods

S1.1 Dataset Preparation

Mini-ImageNet. The Mini-ImageNet dataset is a sub-dataset of ImageNet, which is widely used in few-shot learning. It selects 100 categories from ImageNet1K. The trainset contains 600 labeled images for each category, a total 60,000 images, and the testset contains 100 labeled images for each category, a total of 10,000 pieces.

CIFAR-100. Each image in CIFAR-100 has two labels: superclass and subclass. There are 500 training images and 100 testing images per subclass. CIFAR-100 has 20 superclasses, and each superclass has 5 subclasses.

Caltech-101. Caltech-101 is an objects image dataset with 101 categories. Approximately 40 to 800 images per category, most categories have around 50 images, 8677 images in total. We divide it into a trainset and a testset according to the ratio of 8:2.

When creating the p-Model datasets, we strive to maintain a consistent frequency of occurrences for each class, while simultaneously varying the combinations of different classes in various orders. For each dataset, we randomly permute the order of all classes, divide them into ten classes, and train on the respective classes to construct p-Models. This approach allows us to generate 10 distinct class models for each dataset. We utilize various random seeds to control the generation of class combinations, ensuring we acquire sufficient p-Models. We randomly selected 150 data from the original training data as the out-of-distribution testset.

For CIFAR-100, it has two classification methods: superclass and subclass. In order to increase the diversity and semantics of p-Model data, we use a more complex way to set up the classes included in each model. (1) The classes trained by each model come from different superclasses. This ensures a wide range of semantic variations. (2) Part of the classes trained by each model come from the same superclass. The selection of these classes is done randomly. (3) The classes trained by each model only come from two different superclasses. In the trainset and testset, we distribute these three division methods in quantity according to 3:2:1.

S1.2 Example of class description from GPT-4

For the word of each class, we use GPT-4 to provide a more detailed and standardized description and definition. Some examples are shown in Table S1. The prompts are:

> "I will give you a list containing various nouns. Please add some short, accurate, and common descriptions to these nouns that can accurately define these nouns, and then return to me a JSON file where the key is the name and the value is the corresponding description. An example of the description is: {"goblet": "a drinking glass with a base and stem", "anemones fish": "live associated with sea anemones", "chiffonier": "a tall elegant chest of drawers"}. The list to be processed is as follows:"

Table S1: **Natural language descriptions of the class names from GPT-4, related to Figures 4 and 6.**

class	description of the class from GPT4
"boy"	"a male child or young man"
"girl"	"a female child or young woman"
"apple"	"a round fruit with red, green, or yellow skin and a crisp, sweet flesh"
"pear"	"a sweet, juicy fruit with a thin skin and a rounded base tapering to a stalk"
"orange"	"a round, juicy citrus fruit with a tough, bright orange rind"

S1.3 Data Preparation for Experiments of Unseen Classes

We divide the 100 classes in CIFAR-100 evenly into two groups/shards. The classes belonging to one group serve as the training model data, while the classes in the other group are intentionally excluded from appearing during the training process. When making these divisions, we take care to distribute categories with similar characteristics into separate groups. For instance, we separate the apple and the orange, both being common fruits, into different groups. Similarly, the bear and the lion, both large carnivorous mammals, are divided, and the boy and the man, both representing the male gender, are also separated accordingly.

Table S2: **Analysis about whether Tina merely memorizes and reproduces parameters, related to Table 1.** The model is CNN, and the dataset is CIFAR-100. We verify Tina on OOD (unseen) tasks. Euclidean distances are calculated to reflect the parameter discrepancies directly. Also, we use model ensemble to verify whether the p-Models generated by Tina are functionally different and have diverse representations. Tina is conditioned on the class names as prompts during training. Here, we showcase two training tasks. "Finetune" refers to the oracle model finetuned on the target personalized dataset, "$\text{Tina}_{\text{name}}$" refers to Tina's generated models during inference prompted on class names, "$\text{Tina}_{\text{des.}}$" refers to Tina's generated models during inference prompted on class descriptions. Average accuracy ("Avg.") refers to the average of individual accuracies. Ensemble accuracy ("Ensemble Acc.") refers to ensembling the four models (1 "Finetune", 2 "$\text{Tina}_{\text{name}}$"s, and 1 "$\text{Tina}_{\text{des.}}$") during inference.

	Individual Acc.						Euclidean Distance		
	Finetune	$\text{Tina}_{\text{name 1}}$	$\text{Tina}_{\text{name 2}}$	$\text{Tina}_{\text{des.}}$	**Avg.**	**Ensemble Acc.**	$\text{Tina}_{\text{name}}$-Finetune	$\text{Tina}_{\text{des.}}$-Finetune	$\text{Tina}_{\text{name}}$-$\text{Tina}_{\text{des.}}$
Task 1	75.3	74.9	74.9	58.9	71.0	**76.2**	4.11	11.41	10.72
Task 2	51.2	51.3	51.0	34.1	46.9	**52.9**	3.41	11.95	11.35

S1.4 Detailed Implementations of Methods

We first train the model on the entire dataset for 50 epochs to obtain a stage-one model.

Classifier Selection: Based on the stage-one model, for each classification task, we only retain the vector representing the corresponding class on the classifier and set the vectors for all other classes to zero.

TAPER: We set up two base models and split the dataset into two shards based on the classification labels. Each base model is initialized using the parameters of the stage-one model and fine-tuned on one of the sharded datasets for 5 epochs. In stage 3, we use the class order of the p-Model in the trainset to train the mixer for 5 epochs, and during the testing phase, the mixer remains frozen.

Tina: For each p-Model data, we initialize it using the parameters of the stage-one generic model as a starting point. At the same time, each class is sequentially reorganized as labels ranging from 0 to 9 for training. We fine-tune the generic model for 10 epochs to obtain the p-Models. For ResNet-20, we only fine-tune the parameters of the classifier, while keeping the remaining network parameters frozen.

S1.5 Hyperparameters

In all experiments, we use the same hyperparameters for training. For the model structure, we set the hidden size to 2048, and the number of the encoder and decoder is 1. Each encoder and decoder has 12 layers, and each self-attention layer has 16 attention heads. For the training process, we divide the model parameters into chunks by layer, and the size of each chunk is 576. We set batch size 64, learning rate $4e^{-4}$, and the gradient clipping coefficient to 0.1.

S1.6 Environments and Resources

All our experiments are conducted on CPU Intel(R) Xeon(R) Silver 4210 CPU @ 2.20GHZ. We employ two Quadro RTX 8000 for data-parallel distributed training. When Tina generates a CNN neural network with 5,000 parameters, each GPU requires 20,000MB of memory, and training for 300 epochs takes approximately 5 hours.

S2 Supplemental Results

Whether Tina Merely Memorizes and Reproduces Parameters. In Table S2, we additionally make an in-depth ablation study about whether Tina merely memorizes and reproduces parameters. The study includes the following aspects.

- **Euclidean Distances:** It is found that the generated models have obvious Euclidean distances from each other and also from the fine-tuned models.
- **Ensemble Learning Ability:** Ensemble learning often demonstrates higher accuracy than individual models, which can be indicative of the diversity in the internal representations of different neural networks, meaning that the manifold representations of the model parameters are not identical. Therefore, we make the generated models and the fine-tuned ones an ensemble to see whether it benefits. The substantial performance boost observed through ensembling Tina-generated models (76.2% vs. 71.0% average) provides further evidence of the structural and functional diversity inherent in the generation process. Since each p-Model is denoised from a distinct random noise seed, Tina's diffusion process explores different regions of the parameter space

that satisfy the same semantic task description. These generated models, while all achieving high individual accuracy, exhibit high complementarity in their internal feature representations. By ensembling these diverse networks, the system effectively mitigates individual model biases and leverages a broader manifold of learned knowledge, confirming that Tina performs generalized mapping rather than simple parameter memorization.

- Taking the above experimental results into consideration, it is evident that Tina is not merely memorizing parameters but generalizing.

Computational Efficiency and Inference Latency. Beyond the diversity and generalization capability of the generated parameters, we also investigated the computational efficiency, which is a critical factor for practical deployment in edge-cloud scenarios. We measured the wall-clock time required to produce a single personalized model (p-Model) on a Tesla V100 GPU. For Tina, this metric represents the inference latency with 1,000 diffusion steps.

As illustrated in Table S3, Tina demonstrates superior efficiency compared to baseline methods. When generating ResNet architectures, Tina achieves an approximate **74× speedup** compared to the traditional *Pretrain+ft* paradigm (which requires extensive fine-tuning time) and is notably faster than TAPER. Even for lighter CNN architectures, Tina maintains a significant speed advantage (4.88s vs. 94.35s for fine-tuning). This orders-of-magnitude reduction in latency confirms that Tina supports highly efficient, on-demand model generation, making it exceptionally well-suited for resource-constrained environments where rapid personalization is required. Beyond inference latency, it is also important to distinguish the one-time training cost of Tina from the cumulative cost of training personalized models separately. Based on our training settings, training a Tina model requires 156 GFLOPs, whereas training a single CNN requires 390 MFLOPs. Although Tina is more expensive than training one individual CNN, Tina is a train-once-for-all model: once trained, it can generate models for the entire combinatorial space of personalized tasks. In contrast, training separate CNNs for all possible task combinations (approximately 1.73×10^{13}) would require about 6.75×10^{12} GFLOPs in total. This comparison highlights the key trade-off of our framework: a larger one-time foundation-model training cost in exchange for dramatically lower total cost when many personalized models are needed.

Table S3: Wall-clock time (s) per p-Model, related to Table 1.

Method	CNN	ResNet
Pretrain+ft	94.35	295.55
TAPER	18.10	22.93
Tina **(Ours)**	**4.88**	**3.99**

Embedding-Space Analysis for Out-of-Distribution Generalization. To further understand why Tina generalizes well to out-of-distribution (OOD) tasks, we analyze whether unseen task descriptions share representation similarities with in-domain ones in a pretrained semantic embedding space. Concretely, we encode the task labels/descriptions from both the training set (*seen*) and the evaluation set (*unseen*) using CLIP text embeddings, and visualize their geometry via t-SNE projection. As shown in Fig. S1, unseen tasks are not isolated into a separate region; instead, they substantially overlap with or appear adjacent to semantically related seen tasks, suggesting that both sets reside on a shared semantic manifold. We additionally annotate several representative neighborhoods to illustrate this semantic continuity (e.g., *willow_tree* and *maple_tree* within the *tree* concept; *woman*, *girl*, and *baby* within *people*; and *castle*, *house*, and *road* within *large man-made outdoor things*). This observation provides an intuitive explanation for Tina's OOD performance: although the tasks are held out, their textual semantics remain continuous with the training distribution under CLIP, allowing Tina to generalize by effectively interpolating within the learned semantic space when mapping task descriptions to generated parameters.

Robustness under Common Corruptions. Beyond standard clean-test evaluation, we further examine the robustness of Tina-generated models under distribution shifts caused by common input corruptions. Specifically, we adopt **CIFAR-100-C**, a widely-used robustness benchmark that applies systematic corruptions (e.g., noise, weather, and compression artifacts) to the CIFAR-100 test set. We compare Tina with the conventional *Pretrain+fine-tune* paradigm, which directly optimizes the target network parameters using task-specific supervised data and thus serves as a natural *upper bound* in this setting. As reported in Table S4, while Tina shows a modest gap on clean data (4.12%$\downarrow$ relative to fine-tuning), the gap under corrupted inputs remains similarly small and does not amplify under noise. Notably, on *Gaussian noise*, Tina is only 3.04%$\downarrow$ compared to fine-tuning, even smaller than the clean-data gap, suggesting that Tina-generated models are not unusually fragile and maintain competitive robustness under perturbations. Importantly, Tina does not exhibit disproportionate degradation under corrupted inputs relative to the Pretrain+fine-tune upper bound. A plausible explanation is that Tina's diffusion-based generation process, which repeatedly denoises parameters from noisy states, may act as an implicit regularizer on the generated parameter distribution. Such iterative denoising may encourage parameter solutions that are more stable to perturbations, which is consistent with the relatively stable performance gap observed across corruption types. We present this as an interpretation rather than a causal claim, but it provides a plausible mechanism for Tina's robustness under common corruptions.

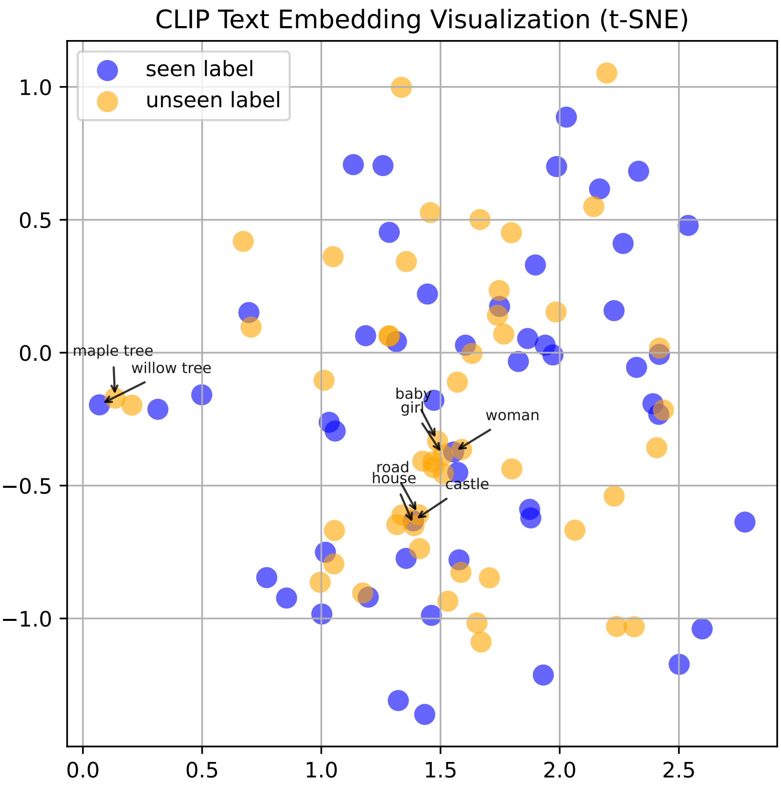

Figure S1: **CLIP text embedding visualization (t-SNE), related to Table 4.** We project CLIP text embeddings of task labels/descriptions into 2D using t-SNE. Blue points denote seen (training) tasks and orange points denote unseen (OOD) tasks. Unseen tasks largely overlap with, or lie close to, semantically related seen tasks (examples annotated), indicating a shared semantic manifold that supports interpolation-based generalization.

S3 Supplemental Discussion: Detailed Related Works

Diffusion models The origin of diffusion models is the study of non-equilibrium thermodynamics. In recent years, DDPM and DDIM have refined diffusion models to a higher level by transforming the paradigm into forward-and-reverse processes in text-to-image generation. Later on, guided-based diffusion models found a better architecture to improve the image generation quality that could beat the GAN-based methods. Then, GLIDE, Imagen, DALL·E 2, and stable diffusion emerged and flourished in the field of image generation and art creation. In the work of diffusion transformer (DiT), the authors found that if the basic architecture of diffusion models is changed to transformers, the scaling law emerges, that scaling the number of parameters can reach the increasing quality of image generation. Based on DiT, in Feb 2024, OpenAI launched Sora, a text-to-video model that can understand and simulate the physical world in motion. In Sora, the DiT architecture is used and scaled to the billions level.

Parameter generation The field of learning to optimize studies how one neural network can learn the update rules (gradients) for optimizing another network. Besides, the studies of hypernetworks focus on how to directly output or modify neural networks' parameters by a hypernetwork. Hypernetworks usually take models' parameters as input and generate parameters, which is different from our paper, which directly maps language space into the parameter space. Hypernetworks were used to generate local models for federated learning, edge-cloud collaboration, few-shot learning, and model editing. A concurrent work ModelGPT also uses text prompts to generate customized models by using large language models as task descriptors. However, ModelGPT didn't target the train-once-for-all personalization scenario, and it uses conventional hypernetwork and meta learning methods while our `Tina` adopts conditional neural network diffusion. Recently, Text-to-LoRA methods map a task description to *LoRA adapters* for a *frozen* large Transformer backbone, enabling instant text-driven specialization in a single forward pass. In contrast, `Tina` focuses on *text-to-model generation*: it produces *standalone* lightweight models (e.g., CNNs) or detachable classifier heads, instead of adapters that still require hosting a heavy backbone at inference time—a key distinction for resource-constrained edge deployment. Additionally, empowered by the strong expressiveness of diffusion models, neural network diffusion was proposed to mimic the optimization rule by diffusion for generating the model parameters. The initial paper is G.pt, which uses DiT to learn to generate the model given a targeted loss or accuracy, and it mimics the optimization process while achieving faster inference compared with vanilla optimization. However, G.pt

Table S4: **Robustness evaluation on CIFAR-100-C, related to Table 1.** Top-1 accuracy (%) of *Pretrain+fine-tune* (upper bound) vs. Tina on clean CIFAR-100 and CIFAR-100-C under representative corruption types (higher is better). The last column reports the relative change of Tina vs. *Pretrain+fine-tune*: $(\texttt{Tina} - FT)/FT$.

**Test Condition**	**Pretrain+fine-tune**	Tina **(Ours)**	**Rel. Δ vs. FT**
Clean CIFAR-100	70.16	67.27	4.12%↓
Gaussian noise	47.73	46.28	3.04%↓
Brightness	67.03	64.49	3.79%↓
Fog	59.34	56.42	4.92%↓
JPEG compression	65.83	62.77	4.65%↓

may have limited use cases; it can only generate the models for the training tasks (i.e., the in-distribution tasks in our paper's terminology), and the accuracies are upper-bounded by the accuracies of checkpoint models in the training datasets. p-diff formally formulates the neural network diffusion problem and proposes to diffuse and generate the batch normalization layers for better accuracies, but the improvement may be marginal, and the diffusion design is not conditioned. It also meets the dilemma of G.pt, which lacks a specific scenario and use case. Recently, GPD uses the diffusion model for few-shot learning in smart city applications, which showcases the applications of neural network diffusion. However, GPD takes the smart city's knowledge graphs as prompts and is tailored for the specific smart city application that cannot be easily extended to other fields. Our Tina takes language texts as prompts, which is more flexible and can be extended to a wider range of applications for the personalization of user demands.

Personalization Instead of training a generic model to provide many users with the same model service, personalization of deep learning models acknowledges users' characteristics and diversity and learns each a customized model. Personalization techniques were introduced in medical AI, recommendation systems, large language models, and especially federated learning. Personalized federated learning studies how to exploit the common knowledge of users and then use it to explore further personalization on users' local datasets under privacy constraints, and techniques like proximal descent, network decoupling, and clustering are used. Recently, the scenario of train-once-for-all personalization was proposed to bridge the gap between edge-side and server-side personalization. Train-once-for-all personalization aims to utilize server-side computation and generic models for fast and effective personalized adaptation to meet the edge users' demands. The original method TAPER finetunes the generic model into several base models and learns MLP-based hypernetworks as mixers to fuse the base models into the personalized one given users' task descriptions. However, the MLP mixer has limited generalization capability, and it cannot be applied to unseen classes, whereas our Tina learns the text-to-model world knowledge and can be generalized to out-of-distribution samples, modalities, and domains.
